# Fatty liver disease in children (MAFLD/PeFLD Type 2): unique classification considerations and challenges

**DOI:** 10.1177/20420188231160388

**Published:** 2023-03-22

**Authors:** Robert Hegarty, Eirini Kyrana, Emer Fitzpatrick, Anil Dhawan

**Affiliations:** Paediatric Liver, GI and Nutrition Centre, and MowatLabs, King’s College Hospital, London, UK; Paediatric Liver, GI and Nutrition Centre, and MowatLabs, King’s College Hospital, London, UK; Department of Gastroenterology, Hepatology and Nutrition, Our Lady’s Hospital Crumlin, Dublin, Ireland; Paediatric Liver, GI and Nutrition Centre, and MowatLabs, King’s College Hospital, London SE5 9RS, UK

**Keywords:** MAFLD, paediatric fatty liver disease, PeFLD

## Abstract

In children, fatty liver disease is a group of disorders that often overlaps with inherited metabolic disorders (IMDs), which requires prompt diagnosis and specific management. Metabolic dysfunction–associated fatty liver disease (MAFLD) or, formerly, non-alcoholic fatty liver disease (NAFLD) is the hepatic component of a multisystemic disease that requires a positive criteria in metabolic dysfunction for diagnosis. However, in children, the diagnosis of MAFLD is one of the exclusions of an IMD [paediatric fatty liver disease (PeFLD) type 1] including the possibility that an IMD can be identified in the future following investigations that may be negative at the time. Therefore, while children with fatty liver with metabolic dysfunction could be classified as MAFLD (PeFLD type 2) and managed that way, those who do not fulfil the criteria for metabolic dysfunction should be considered separately bearing in mind the possibility of identifying a yet undiagnosed IMD (PeFLD type 3). This concept is ever more important in a world where MAFLD is the most common cause of liver disease in children and adolescents in whom about 7% are affected. The disease is only partially understood, and awareness is still lacking outside hepatology and gastroenterology. Despite its increasing pervasiveness, the management is far from a one-size-fits-all. Increasing complexities around the genetic, epigenetic, non-invasive modalities of assessment, psychosocial impacts, therapeutics, and natural history of the disease have meant that an individualised approach is required. This is where the challenge lies so that children with fatty liver are considered on their own merits. The purpose of this review is to give a clinical perspective of fatty liver disease in children with relevance to metabolic dysfunction.

## Introduction

There has been widespread endorsement of the new terminology, metabolic dysfunction–associated fatty liver disease (MAFLD), formerly known as non-alcoholic fatty liver disease (NAFLD), since its introduction in 2020.^1^ MAFLD represents the hepatic component of a multisystemic disease including type 2 diabetes mellitus (T2DM), cardiovascular disease and chronic kidney disease.^2^ The intention for the renaming of NAFLD to MAFLD was to provide a positive diagnosis given the increasing understanding of the pathomechanism that involves systemic, metabolic dysfunction.^3^ Importantly, it also provides a unified terminology for a more accurate classification (e.g. International Classification of Diseases) to enhance the legitimacy of clinical practice and research.^3^ However, children with fatty liver merit special attention due to the risk of missing an underlying genetic defect with specific therapeutic implications. This is particularly important during an obesity epidemic where there is more opportunity for misdiagnosis in children with fatty liver highlighted in this review and a patient story (*Case report 1*). This article reviews the clinical challenges and considerations that children with fatty liver face in this era. For the purposes of this discussion, we will be using the ‘MAFLD’ terminology for children with paediatric fatty liver disease (PeFLD) type 2.

## NAFLD to MAFLD (PeFLD type 2) in children

**Figure 1.**
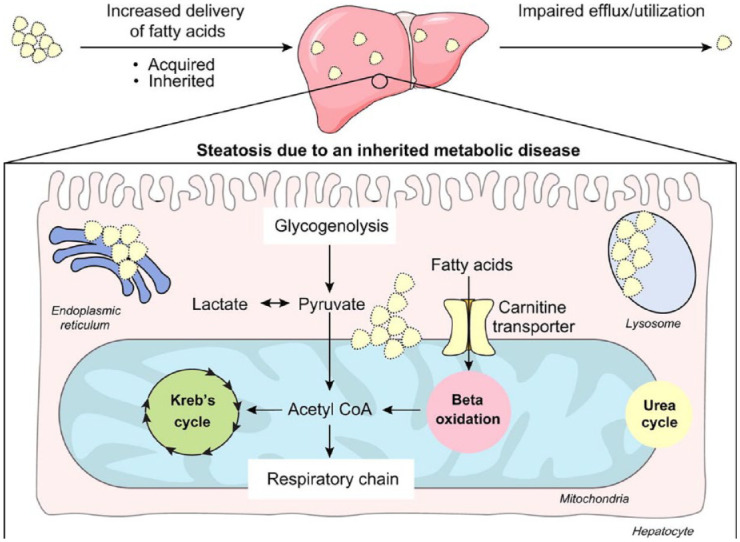
Accumulation of fat droplets in paediatric fatty liver disease: a schematic representation. Build-up of fat in hepatocytes can be due to (1) increase in delivery of fatty acids and cholesterol (e.g. dietary intake), (2) organelle dysfunction in inherited metabolic disease or (3) impaired efflux of fat (e.g. lysosomal acid lipase deficiency). Adopted from Hegarty *et al*.^4^

**Figure 2.**
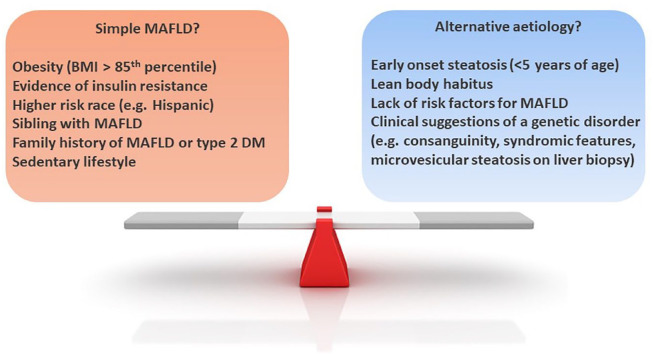
Evaluating the risk factors for metabolic dysfunction–associated fatty liver disease in paediatric fatty liver disease in children. Adopted from Hegarty *et al*.^4^

Children with fatty liver require their own consideration when it comes to the differential diagnosis and diagnostic work up for fatty liver. There are a wide variety of inherited metabolic disorders (IMDs) that can cause fatty liver and mimic or coexist with MAFLD.^4^ In an audit carried out at King’s College Hospital looking at all conditions that cause steatosis in the liver as many as 178 of 441 children had a diagnosis of an IMD^5^ (*Case report*). With increasing prevalence of fatty liver in children, there is a risk that children are falsely labelled as MAFLD when, in fact, the underlying reason for the fatty liver is an IMD. Furthermore, the list of such IMDs are increasing, including congenital disorders of glycosylation^6^ and aminoacyl transfer RNA synthetase deficiencies^7^ (Figure 1). Clues that may point towards an underlying IMD include younger age (<5 years) and lean body habitus^4^ (Figure 2). Indeed, the introduction of MAFLD as a new definition has been welcomed as a positive, diagnostic criteria will reduce the chances of such diagnostic errors.^5^ However, the diagnosis for one reason for fatty liver does not exclude another. Moreover, the paediatricians who care for children with these conditions may work in contrasting fields and not be fully aware of the other: a specialist in IMD *versus* a generalist looking after children with MAFLD. It is, therefore, ever more important to raise awareness relating to the complexity of fatty liver in children and the need for accurate diagnosis.

We suggest a nomenclature that considers the different aetiologies of fatty liver in children.^5,8^ Namely, PeFLD with the following subtypes: type 1, those diagnosed with an IMD; type 2, those with metabolic dysfunction, that is, MAFLD; type 3, fatty liver without an identifiable cause. Those that fit into type 1 and type 2 PeFLD have a positive diagnosis. The reason for a third subtype of unclassified children with fatty liver is to keep the diagnosis open to the possibility of a potential IMD that may be revealed in the future without risking the child being mislabelled as ‘fatty liver’.

The diagnosis of MAFLD in adults stipulates evidence of hepatic steatosis, in addition to one of the following: (1) overweight/obesity, (2) presence of T2DM or (3) evidence of metabolic dysregulation.^8^

Hepatic steatosis can be suspected either by imaging, serum biomarkers or histological assessment. The new definition invites the use of ultrasound scan, the most common modality to detect hepatic steatosis while recognising its limitations such as low diagnostic accuracy in mild steatosis^9^ – in line with a consensus statement by the European Society of Gastroenterology, Hepatology and Nutrition.^10^

Obesity in adults is defined as a body mass index (BMI) ⩾ 25 kg/m^2^ in Caucasians or BMI ⩾ 23 kg/m^2^ in Asians.^3^ In children, body composition varies with age and sex, so comparisons must be made to an appropriate population of the same sex and age by standard deviation or *Z*-scores. Many countries as well as specific conditions (e.g. Down’s syndrome) have their own growth charts.^11,12^ Therefore, there is no single absolute value of BMI that defines obesity in children and the World Health Organization (WHO), Centers of Disease Control and Prevention, and International Obesity Task Force each has definitions for obesity using BMI centiles according to age groups.^13–15^

In terms of diagnosing T2DM, the definition in adults of a random venous plasma glucose concentration ⩾ 11.1 mmol/l or a fasting plasma glucose concentration ⩾ 7.0 mmol/l (whole blood ⩾ 6.1 mmol/l) has been adopted in children. However, a consistent criterion to define metabolic syndrome (MS) in childhood and adolescence is lacking.^16,17^ The definition and prevalence of MS depends on the age and the diagnostic criteria used (Table 1). In a study of 217 obese children and adolescents aged 8–15 years, MS defined by the International Diabetes Federation (IDF) and WHO gave a prevalence of 43% and 55%, respectively.^18^ In another study, the prevalence of MS according to the IDF definition provided the lowest prevalence (0.3–9.5%), whereas the classification of de Ferranti *et al.* yielded the highest (4.0–26.4%).^19^ The high rate of reversal of impaired glucose tolerance in children and adolescents during puberty, including a 66% conversation rate to normal glucose metabolism, also ought to be taken into account.^20^ Furthermore, metabolic dysfunction related to monogenic or mitochondrial diabetes should be considered in children who have associated features of another systemic illness or those who do not develop ketones during episodes of hyperglycaemia.^21^ Finally, non-obese individuals with impaired glucose tolerance as a biologically distinct phenotype with a stronger genetic propensity for fatty liver need to be considered.^22^

**Table 1.** Different definitions of metabolic syndrome in children.

Criteria	Cook *et al.* 12- to 19-year-olds	De Ferranti *et al*.	Alberti *et al*. 10- to 16-year-olds	Eslam *et al.*
	⩾3 criteria	⩾3 criteria	Obesity and at least 2 of 4 remaining criteria	Overweight/obesity, T2DM or ⩾2 of below criteria in lean/normal weight
WC	WC ⩾ 90th percentile	> 75th percentile for age and sex	WC ⩾ 90th percentile or adult cut-off if lower	WC ⩾ 102/88 cm in Caucasian men and women (or ⩾ 90/80 cm in Asian men and women)
Impaired glucose tolerance	⩾ 110 mg/dl	⩾ 6.1 mmol/l (⩾110 mg/dl)	⩾ 5.6 mmol/dl (100 mg/dl) or known T2DM	Prediabetes^a^
Hypertriglyceridaemia	⩾ 110 mg/dl	⩾ 1.1 mmol/l (⩾100 mg/dl)	⩾ 1.7 mmol/l (⩾150 mg/dl)	Plasma triglycerides ⩾ 150 mg/dl (⩾1.70 mmol/l)^b^
Low HDL	HDL ⩽ 40 mg/dl	HDL < 1.3 mmol/l (⩽ 50 mg/dl) (boys aged 15–19 years, < 1.17 mmol/l)	HDL < 1.03 mmol/l (⩽ 40 mg/dl)	HDL-cholesterol < 40 mg/dl (<1.0 mmol/l) for men and < 50 mg/dl (<1.3 mmol/l) for women^b^
Hypertension	⩾ 90th percentile	> 90th percentile for age, sex and height	SBP ⩾ 130 / DBP ⩾ 85 mmHg	⩾ 130/85 mmHg^b^
Homeostasis model assessment of insulin resistance score	NA	NA	NA	⩾ 2.5
Plasma high-sensitivity C-reactive protein level	NA	NA	NA	⩾ 2mg/l

DBP, diastolic blood pressure; HDL, high-density lipoprotein; NA, not applicable; SBP, systolic blood pressure; T2DM, type 2 diabetes mellitus; WC, waist circumference.

a Defined as fasting glucose levels 100–125 mg/dl (5.6–6.9 mmol/l) or 2-h post-load glucose levels 140–199 mg/dl (7.8–11.0 mmol/l) or HbA1c 5.7–6.4% (39–47 mmol/mol).

b Or any specific drug treatment.

Despite these discordances that may be encountered in defining MAFLD in children compared with adults, some recent studies have assessed the utility of the new definition in predicting those who may have more advanced disease. For instance, in a cohort of 954 obese children and adolescents, the presence of metabolic dysfunction correlated with higher cardiovascular risk in comparison with those who were obese with fatty liver but without the metabolic dysfunction.^23^ However, in another cross-sectional study from the United States looking at 12- to 18-year-olds with fatty liver, the prevalence of advanced liver fibrosis did not differ significantly according to whether they met the MAFLD criteria.^24^

Taken together, longitudinal studies are still required to help understand the natural history of obesity and MS in children that are age, sex and ethnicity specific. In time, the definition of MS in children may change as will its application to MAFLD.

**Case report**

A previously fit and healthy 3-year-old girl was referred to paediatric hepatology with a history of abnormal liver function tests and hepatomegaly on clinical examination detected following hospital attendance due to an upper respiratory tract illness. The liver function tests were ALT 112 IU/l, AST 157 IU/l, GGT 15 IU/l and INR 0.9. Magnetic resonance imaging of the liver demonstrated an enlarged liver with diffuse fatty infiltration. Diagnostic investigations focussed on an IMD associated with fatty liver / PeFLD type 1 given the young age. Molecular genetic testing revealed two pathogenic, *in trans*, variants in *ALDOB*. The child received a diagnosis of hereditary fructose intolerance (HFI), and a dietary management plan was instituted prior to the patient becoming acutely symptomatic. Symptoms of HFI can be subtle as it is dependent on the individual’s residual enzyme activity and dietary fructose exposure (1) and fatty liver is a common finding in treated individuals (2).

## Epidemiology

MAFLD or, formerly, NAFLD is the most common chronic liver disease in children. The pooled mean prevalence of fatty liver disease from a meta-analysis carried out in children and young adults in 2022 was 7.4% in the general population and as high as 52% in the obese.^25^ Prevalence is higher in males than females with an odds ratio of 1.63 in the general population and 2.02 in the obese.^26^ However, many of these studies used alanine aminotransferase (ALT) with a threshold of 40 U/l as a method for screening for fatty liver which has a lower sensitivity for detecting fatty liver when compared with imaging.^26^ Therefore, under the new definition of MAFLD, which requires identification of hepatic steatosis by biopsy, imaging or blood biomarker,^3^ there is likely to be a more stringent inclusion criteria in identifying children with MAFLD. This may lead to an increase in prevalence estimates if only studies that identified hepatic steatosis by imaging or blood biomarker were used.

## Genetics

Several single nucleotide polymorphisms (SNPs) have been demonstrated to increase the susceptibility to fatty liver, including patatin-like phospholipase domain-containing protein 3 (*PNPLA3*) Iso148Met (rs738409)^27^; transmembrane 6 superfamily member 2 (*TM6SF2*) Glu167Lys (rs58542926)^28^; glucokinase regulator (*GCKR*) Pro446Leu (rs1260326)^29^; and membrane bound o-acyltransferase domain containing 7 (*MBOAT7*) Gly17Glu (rs641738).^30^ The combined effects of these variants have also shown to confer significantly higher risks of cirrhosis and hepatocellular carcinoma (HCC).^31^ On the contrary, some variants are protective: hydroxysteroid 17-beta dehydrogenase 13 (*HSD17B13*) TA splice variant rs72613567,^32^ mitochondrial amidoxime reducing component 1 (*MTARC1*) and *HSD17B13* variants reduce severity of MAFLD in children. The combination of these susceptibility and protective alleles contribute to the genetic component – ‘GenComp’ – of a genetic risk score.^33^ The genetic predisposition may be considered the first hit and insulin resistance as the second.^34^

Studies focussing specifically on children are still lacking. A study involving 234 Hispanic boys from the Nonalcoholic Steatohepatitis Clinical Research Network identified SNPs associated with the NAFLD activity score (NAS) on chromosome 8 in the trafficking protein particle complex 9 (TRAPPC9) as well as a region close to actin related protein 5 associated with fibrosis.^35^ In the same study cohort, 10 SNPs associated with obesity and 9 with insulin resistance, HOMA-IR and HbA1c, were identified.^36^ A further novel locus associated with fasting insulin was identified in *CSMD1* on chromosome 8 in a cohort of Chilean adolescents.^37^ In another study based on 228 children with fatty liver, *PNPLA3* (rs738409), *TM6SF2* (rs58542926) and *SAMM50* (rs2073080 and rs3761472) were identified as independent risk factors.^38^

Identifying individuals at genetic risk in the early years will provide an opportunity for early intervention. However, there are still many unknowns such as whether these risk alleles are associated with histological severity, younger age of onset or driver for disease progression in children. The difficulty will lie in interpreting this emerging evidence and incorporating it into clinical practice.

## Epigenetics

The perinatal period is a critical time in development, and the intrauterine environment has been shown to confer long-lasting influences on cardiometabolic diseases.

The effects of maternal overnutrition on their offspring has been the focus of numerous studies. It has been shown that children born to diabetic or obese mothers have increased subcutaneous fat at birth^39^ and are at increased risk of developing MS in childhood as early as 6 years of age.^40^ In a postmortem study of stillborn infants, those born to mothers with gestational diabetes had a prevalence of 79% steatosis *versus* the 17% in those born to mothers who were not diabetic.^41^ The severity of steatosis positively correlated with foetal body weight and gestational age independent of maternal obesity, implying an increase in hepatic fat with prolonged exposure to the diabetic environment.^41^ In another study using magnetic resonance imaging (MRI), intrahepatic lipid content was higher in infants of mothers who were obese and those with type 2 diabetes than in those born to normal-weight mothers.^42^ Maternal diabetes in pregnancy, therefore, creates an unfavourable environment associated with hepatic steatosis and MS. However, whether hepatic steatosis in infancy persists to become MAFLD or disappears is unknown. If the origins of MAFLD are indeed *in utero* and persists, the prevalence of MAFLD may be higher than estimated in younger children who are asymptomatic.^43^

Permanent changes in organ function by intrauterine programming occur following *in utero* insults. Both small for gestational age and large for gestational age infants are overrepresented in those who develop fatty liver during childhood and adolescence.^44,45^ Placental insufficiency has also been associated with a higher inflammatory milieu with impairment of glucose tolerance and the developmental of hepatic steatosis.^46^

## Assessment

Detection of steatosis by imaging techniques is the hallmark of diagnosis of fatty liver, and the new definition of MAFLD invites the use of ultrasound scan given its wide availability. Ultrasound can detect > 30% steatosis which is around the level at which it is most clinically significant.^47^ However, it must be recognised that qualitative grades, labelled mild, moderate and severe, may not be accurate^48,49^ and cannot differentiate steatosis to steatohepatitis or fibrosis, which are the main determinants of outcome. Therefore, ultrasound results must be interpreted with caution.

Increasingly, alternative, non-invasive modalities of assessment of hepatic steatosis are being used. In controlled attenuation pressure (CAP), which is based on ultrasound signals acquired by transient elastography (TE) or Fibroscan® (Echosens SA, Paris, France), detection of 10–30% steatosis is possible alongside fibrosis based on the principle of attenuation of ultrasound signal through fat.^50^ Optimal cut-offs were 248, 268 and 280 dB/m for the detection of steatosis grading 0, 1 and 2 using the M probe bearing in mind this can be influenced by aetiology of the disease and presence or absence of obesity.^51,52^ It must be noted that grading of steatosis was not accurate enough using the XL probe that is recommended for obese patients.^53^ In the assessment of fibrosis in 52 children with fatty liver undergoing liver biopsy, TE values of 5, 7 and 9 kPa predicted ‘any’ fibrosis, significant fibrosis and advanced fibrosis, respectively, with excellent performance.^54^

Point quantification shear wave elastography (pSWE) is another ultrasound-based technology that involves acoustic radiation force impulse (ARFI). The diagnostic performance in staging fibrosis in fatty liver patients was comparable with TE for fibrosis stages 3 and 4 but underperformed at stage 2.^55^

On the contrary, magnetic resonance (MR) imaging has shown high diagnostic accuracy.^56^ In the largest paediatric study, MR-estimated liver proton density fat fraction (PDFF) correlated well with histological steatosis grade.^57^ However, its use including MR spectroscopy, the imaging gold standard, as well as multiparametric MR^58^ and MR elastography^59^ is limited by availability and is more likely restricted for use in the research setting. Future challenges will be around the validation of these studies in children before integration into clinical practice.

Liver histology has traditionally been the gold standard for the assessment of steatosis. However, it is still subject to sampling bias and inter- and intra-examiner variability let alone its invasive nature and possibility of complications. Grading is carried out according to the proportion of hepatocytes containing fat macrovesicles on hematoxylin and eosin staining (grade 0, < 5%; grade 1, 5–33%; grade 2, 34–66%; and grade 3, >66%).^60^ In terms of the prevalence, evaluation of liver histology from 742 children from those undergoing autopsy for unexpected deaths demonstrated that 9% had fatty liver and 3% steatohepatitis.^61^ When considering children undergoing initial assessment for fatty liver, there is a high prevalence of advanced disease at presentation: 10–25% have advanced fibrosis at initial presentation and 25–50% have steatohepatitis.^62^ In children, inflammation is often portal based, steatosis may be periportal in distribution, located in acinar zone 3 or panacinar while ballooning is uncommon [type 2 non-alcoholic steatohepatitis (NASH)].^63^ In adults, acinar zone 3 is typically where accumulation of fat is seen and where fibrosis begins. The progressive form of steatohepatitis features these steatotic hepatocytes with lobular inflammation and cell injury in the form of hepatocyte ballooning and inflammation (type 1 NASH), whereby ballooning denotes a pattern of liver cell injury of cytoplasmic swelling and rounding.^64^ This suggests that there may be a different disease process or reflects the differences in the maturing process between adults and children. However, without sequential biopsies over time, it is difficult to tell the relevance of cross-sectional snap shots in an evolving, chronic process. In the meantime, questions around how often and in whom biopsies should be performed to monitor disease progression remains unanswered.

Numerous non-invasive fibrosis scores of fatty liver have been studied in adults, including aspartate aminotransferase (AST) to ALT ratio, AST to platelet ratio index (APRI), NAFLD fibrosis score and Fib-4 Index. However, they remain unvalidated in children.^65^ The Paediatric NAFLD Fibrosis Index (PNFI) based on age, waist circumference and triglycerides predicted advanced fibrosis with an area under the curve (AUC) of 0.74,^66^ but these results have not been replicated elsewhere.^67,68^

Several serum biomarkers of liver injury and extracellular matrix turnover, including cytokeratin 18 (CK-18), hyaluronic acid and enhanced liver fibrosis (ELF), have been studied in children. CK-18 including the antigens M30 and M65 are released by hepatocytes upon apoptosis. It has been extensively studied in adults and demonstrated to be useful in distinguishing children with steatohepatitis against simple steatosis as well as those with significant fibrosis against no/minimal fibrosis.^69^ When CK-18 was measured over time in 152 children, a greater decrease in levels was observed in those with histological improvement in steatohepatitis.^70^ When 30 studies were investigated in a meta-analysis in 2020, M65 performed better than M30 and AUCs were 0.82 for steatohepatitis; 0.68 (M30) for significant (F2–F4) fibrosis; and 0.75 (M30) for advanced (F3–F4) fibrosis.^71^ Hyaluronic acid has been shown to predict liver fibrosis in children with fatty liver in single-centre studies, but cut-off values varied widely when these studies are compared.^72–74^ Similarly, heterogeneous cut-off values are observed when the ELF test which incorporates the use of hyaluronic acid, tissue inhibitor of metalloproteinases 1 (TIMP-1) and amino-terminal propeptide of type III procollagen (PIIINP) was employed to predict fibrosis stage in 111 children.^68,75^ However, recent evidence using data from the Treatment of NAFLD in Children (TONIC) trial suggest that dynamic changes in serum ALT and gamma glutamyl-transferase (GGT) are strongly associated with change in liver histology.^76^

Despite these numerous efforts to identify biomarkers for liver fibrosis in fatty liver, most of these paediatric studies are single, tertiary centre studies with highly selective patients. Therefore, without further validation, its applicability to the wider population is questionable and may be the reason for the lack of reproducibility. These issues should be tackled in the face of adapting MAFLD as a new definition.

Other aetiologies that require consideration when evaluating a child with MAFLD include use of parenteral nutrition and several medications (corticosteroids, antidepressants, HIV antiretroviral therapy among others), malnutrition, coeliac disease, endocrinopathies (e.g. hypothyroidism), viral hepatitis (i.e. hepatitis C genotype 3) and autoimmune hepatitis in addition to IMDs.^77,78^

## Complications

The most relevant and important hepatic complication of MAFLD is fibrosis stage. Approximately, 11–15% of children will have advanced fibrosis at presentation when referred to a paediatric gastroenterologist for evaluation of fatty liver.^79,80^ However, figures are much higher in the obese.^81^ Over a mean follow-up of 1.4 years, fibrosis improved in 34% of the children but worsened in 23% while bridging fibrosis remained unchanged at 15%.^79^ Rapid progression to cirrhosis over 1–2 years has been reported, but this is in isolated cases.^82,83^ There are a few reports of liver transplantation in young adulthood in the follow-up of children with fatty liver.^79,84^ Inevitably, these patients with cirrhosis secondary to fatty liver are at risk of developing HCC like patients with cirrhosis from other aetiologies, but to our knowledge, this has not been reported in children.

Sarcopenia is a condition characterised by loss of skeletal muscle mass and function and has been identified in multiple chronic diseases.^85^ Recent studies in adults have demonstrated that the presence of sarcopenia in patients with fatty liver disease is associated with a higher likelihood of having steatohepatitis and advanced liver fibrosis independent of other confounding factors such as age, sex, BMI and insulin resistance.^86^ Patients with sarcopenia have a 2- to 5-fold increase to have a fatty liver^87–90^ and a 2.5-fold increase in steatohepatitis and significant fibrosis if they had a fatty liver.^91^ In the study by Koo *et al.*,^88^ in 309 subjects with biopsy proven fatty liver disease, the prevalence of sarcopenia in subjects without fatty liver disease, with fatty liver disease and with steatohepatitis were 8.7%, 17.9% and 35.0%, respectively. In children, there is one retrospective study showing that fatty liver disease (as assessed by ultrasonography) was significantly associated with relatively low skeletal mass in non-obese children and adolescents.^92^ From a mechanistic viewpoint, insulin resistance is associated with the development of MAFLD,^93^ but it also exacerbates muscle catabolism as insulin is an anabolic hormone.^94^ There is also systemic inflammation which increases muscle proteolysis^95^ and contributes to the development of MAFLD.^96^ The above findings mostly from adult studies are significant because they highlight the important role of increasing muscle mass *via* exercise and resistance training as part of a treatment strategy for fatty liver disease potentially in children. Elucidating the specific molecular pathways involved may allow for novel treatment targets to be identified, but clearly there is more need for research in this area in paediatrics.

Another important yet underexplored aspect of MAFLD is how mental health is linked to disease status. The prevalence of depression and anxiety in children, young people and adults with obesity is high.^97–99^ In a survey of 239 children who were a part of the NASH Clinical Research Network, children with fatty liver disease had worse total, physical and psychological quality of life scores with fatigue, trouble sleeping and sadness accounting for this difference with healthy children.^100^ Cognitive-behavioural therapy and counselling have been used with success in adults.^101,102^ However, more work is required in children to break the cycle of mental health as a driver of MAFLD and vice versa.

## Management

Lifestyle change resulting in weight loss prior to the onset of advanced fibrosis is the mainstay of management. Under a 12-month programme with diet and physical exercise leading to an average weight loss of 4.9 kg, significant reductions in the level of fasting glucose, insulin, lipids, liver enzymes and liver echogenicity on ultrasonography was possible.^103^ Similar benefits were demonstrated when a multidisciplinary programme of dietary and exercise advice was employed in a paediatric liver, sub-specialty clinic.^104^ However, there are no detailed recommendations on the kind or amount of exercises or diet that are required to achieve histological improvement. Both studies had a high attrition rate highlighting the challenges inherent to weight management interventions. Stages of Change Readiness and Treatment Eagerness Scale (SOCRATES) questionnaires from 41 children in a hepatology clinic showed that there is low recognition of their obesity as a problem and a desire to change.^105^

The TONIC trial evaluated the effect of metformin and vitamin E on ALT levels and histological improvement.^106^ There was no difference in the primary outcome of ALT reduction between the placebo and vitamin E or metformin treatment groups. However, vitamin E showed benefits in terms of improvements in histology, including ballooning, MAFLD activity and the proportion that resolved steatohepatitis at 96 weeks. The American Association for the Study of Liver Diseases (AASLD) advises consideration of use of vitamin E in children with the risks and benefits explained; metformin at 500 mg twice daily is not recommended specifically to treat fatty liver, but higher doses and its effects on fatty liver and metabolic dysfunction should be further evaluated.^107^

Docosahexaenoic acid (DHA) and eicosapentaenoic acid are omega-3 fatty acids that are recommended in adults for hypertriglyceridemia.^107^ In children, a recent meta-analysis of six randomised placebo–control trials looking at the use of omega-3 fatty acids in fatty liver disease showed improvements in transaminases, hepatic steatosis and BMI.^108^ However, these effects may have been confounded by the lifestyle interventions recommended in the studies.

Obesity is associated with a lower diversity of gut microbiota, and probiotics are an alternative way to attempt to re-establish a healthy diversity of flora. In a meta-analysis of nine randomised trials with fatty liver disease, the probiotic therapy group has significant reduction in the levels of serum AST, ALT and total cholesterol in comparison with the control group.^109^ In a study of 20 obese children who received *Lactobacillus rhamnosus* strain GG for 8 weeks, there was an improvement in ALT in comparison with the control group.^110^ Similar findings were demonstrated in a study of 64 obese children in addition to improvements in cholesterol, low-density lipoprotein-C triglycerides and waist circumference decrease.^111^

The European Association for the Study of the Liver (EASL), European Association for the Study of Diabetes (EASD) and European Association for the Study of Obesity (EASO) and AASLD guidelines provide recommendations for treatment of fatty liver disease. Lifestyle modifications is the first line of treatment. However, there is no single intervention that has shown to improve fibrosis in children. Vitamin E, omega-3 fatty acids and probiotics have shown beneficial effects to a mixed degree in terms of improvements in ALT, steatosis on imaging and histology, but these guidelines do not recommend their use universally due to the lack of biopsy endpoints and long-term evidence.^77,107^

## Conclusion

The term NAFLD was described 40 years ago based on the exclusion of excess alcohol for diagnosis. Paediatricians adopted the terminology even though alcoholism is not characteristic of children. Since then, fatty liver disease has grown to be the leading cause of chronic liver disease in children. The renaming of NAFLD to MAFLD has been widely endorsed, and children must not be left behind in this global movement. However, there are some fundamental challenges including adopting an agreed definition of metabolic dysfunction in children. Furthermore, the increasing complexity of MAFLD in children has meant that there is a risk that individualised approaches could be lost in the face of increasing prevalence. In this regard, our message has been to use the terminology paediatric fatty liver disease or PeFLD until a positive diagnosis such as MAFLD is made. Paediatricians need to be aware of these facts and embrace the diagnostic and treatment challenges of MAFLD to reverse the course of the disease in childhood.

## Declarations

**Ethics approval and consent to participate:** Not applicable.

**Consent for publication:** Written informed consent to publish the *case report* was obtained from the patient’s next of kin.

**Availability of data and materials:** Not applicable.
